# mSphere of Influence: Metabolic redundancies enhance pathogenesis

**DOI:** 10.1128/msphere.00239-24

**Published:** 2024-07-03

**Authors:** McKenzie K. Lehman

**Affiliations:** 1 Department of Pathology, Microbiology and Immunology, University of Nebraska Medical Center, Omaha, Nebraska, USA; University of Michigan, Ann Arbor, Michigan, USA

**Keywords:** metabolism, amino acid transport, *Staphylococcus aureus*

## Abstract

McKenzie Lehman works in the field of bacterial pathogenesis and metabolism. In this mSphere of Influence article, she reflects on how three papers entitled “Glycolytic dependency of high-level nitric oxide resistance and virulence in *Staphylococcus aureus”* by N. P. Vitko, N. A. Spahich, and A. R. Richardson (mBio 6:e00045-15, 2015, https://doi.org/10.1128/mbio.00045-15), “The *Staphylococcus aureus* cystine transporters TcyABC and TcyP facilitate nutrient sulfur acquisition during infection” by J. M. Lensmire, J. P. Dodson, B. Y. Hsueh, M. R. Wischer, et al. (Infect Immun 88:e00690-19, 2020, https://doi.org/10.1128/iai.00690-19), and “The second messenger c-di-AMP inhibits the osmolyte uptake system OpuC in *Staphylococcus aureus*” by C. F. Schuster, L. E. Bellows, T. Tosi, I. Campeotto, et al. (Sci Signal 16:ra81, 2016, https://doi.org/10.1126/scisignal.aaf7279) impacted her work on bacterial metabolism and pathogenesis.

## COMMENTARY

*Staphylococcus aureus* is a crafty pathogen that has the ability to asymptomatically colonize the host, but also infect nearly every organ system. The virulence of *S. aureus* is in contrast to many other species of staphylococci (e.g., *Staphylococcus epidermidis, Staphylococcus haemolyticus, Staphylococcus hominis,* etc.), which also colonize the skin, but cause disease far less frequently. This difference in virulence is attributed to the myriad of virulence factors encoded by *S. aureus* and its robust metabolic capacity and nimble regulatory network that allows the organism to not only survive but thrive under various nutrient limitations. The focus on metabolism as an important factor that mediates *S. aureus* pathogenicity is represented in a multitude of papers in the last 20 years [reviewed in references (1–4)]. It is this body of literature, including “Glycolytic dependency of high-level nitric oxide resistance and virulence in *Staphylococcus aureus”* by Vitko et al. (5), “The *Staphylococcus aureus* cystine transporters TcyABC and TcyP facilitate nutrient sulfur acquisition during infection” by Lensmire et al. (6), and “The second messenger c-di-AMP inhibits the osmolyte uptake system OpuC in *Staphylococcus aureus*” by Schuster et al. (7), that have shaped my current research that aims to understand the spatiotemporal nutrients utilized by *S. aureus* during an infection by studying nutrient acquisition systems and their impact on pathogenesis.

The elegant work by Vitko et al. (5) demonstrated that glucose catabolism via glycolysis is required for *S. aureus* survival in macrophages and during the establishment of both the murine bacteremia and skin and soft tissue models of infection. This finding has important implications for gene regulation during an infection as the carbon catabolite repressor, CcpA, is active when glucose is present and represses many metabolic pathways, including those for amino acid catabolism (8–11). This proved an important observation during my characterization of the proline transporters encoded by *S. aureus* (12). In these studies, we found that proline import, but not biosynthesis, was imperative for the establishment of both the bacteremia and skin and soft tissue murine models of infection. Furthermore, Vikto et al. (5) illustrated that redundancy in metabolic processes enhanced virulence of *S. aureus*. Specifically, *S. aureus,* but not other staphylococci, withstood nitric oxide (NO^.^) stress, including during survival in macrophages, by encoding an additional NO^.^-responsive lactate dehydrogenase (13). Additional work by Vitko and colleagues revealed that *S. aureus* also encodes an expanded repertoire of glucose transporters compared to other staphylococci that further enhances its fitness in a murine model of skin and soft tissue infection (14).

There is also evidence that the redundancy in metabolic processes encoded by *S. aureus* allows the pathogen to colonize different niches. For example, *S. aureus* has a branched respiratory chain, with one terminal oxidase required for heart colonization and the other for liver colonization (15). Using genetic screens and differential growth assays, Lensmire and colleagues identified that *S. aureus* encodes two cystine transporters TcyABC and TcyP that facilitate nutrient sulfur import and have differential effects on organ colonization (6). In many cases, TcyABC and TcyP appear to be functionally redundant, importing a variety of sulfur-containing molecules (cysteine, cystine, *N-*acetyl cysteine), yet TcyABC was found to be the sole transporter of homocystine. Fitness analysis in a murine bacteremia model revealed differential effects of the cystine transporter mutants, with TcyP appearing important for liver colonization, whereas both TcyP and TcyABC were required for heart colonization. Additional studies from the Hammer laboratory revealed that *S. aureus* also encodes a glutathione import system to acquire nutrient sulfur and this system provides a competitive growth advantage over other staphylococcal species (16). These data reveal the breadth of redundancy for nutrient sulfur acquisition encoded by *S. aureus* and provide clues to potential differences in sulfur-source availability in different niches of the host during an infection. In my analysis of proline transporters encoded by *S. aureus,* I, too, found redundancy, with ProT and PutP identified as the primary proline transporters (12). In murine bacteremia and skin and soft tissue models of infection, proline transport was required for establishing an infection, with either transporter sufficient to support an infection. Yet, it appears as though ProT is the sole proline transporter required for acquiring proline under sodium chloride-mediated osmotic stress. This is another example of the redundancy found in *S. aureus* metabolic processes and we are just beginning to understand its impact on the pathogenesis of the organism.

Proline, in addition to being a proteinogenic substrate and carbon source, is also an important osmolyte. There was initial speculation that *S. aureus* encoded multiple proline transporters, including transporters important for responding to osmotic stress (17–21). The experiments by Schuster et al. (7) revealed that one of the putative proline transporters, OpuC, is actually a carnitine transporter that is regulated by the second messenger cyclic-di-AMP. Cyclic-di-AMP directly binds to and represses a variety of osmolyte transporters, including the potassium transporters Ktr and Kdp (22). There is also evidence that the osmolyte transporter OpuD and glutamine transporter AlsT are also responsive to cyclic-di-AMP levels (23, 24). These data, in addition to the observation that altered pools of cyclic-di-AMP affects growth in rich media (23) and antibiotic sensitivity (25), indicate that understanding the interplay of cyclic-di-AMP signaling, osmolyte/metabolite transport, and metabolism is important for understanding *S. aureus* pathogenesis and the development of future therapies.

We are just beginning to understand the implications of the various observed redundancies on the metabolic capacity and pathogenesis of *S. aureus*. The data we currently have available in the literature suggest that an expanded repertoire of metabolic processes results in a fitness advantage of *S. aureus* over closely related staphylococcal species under a variety of growth conditions. These papers have influenced the way I think about the experiments I design and how to interpret the data to better understand the evolution of *S. aureus* as a pathogen in relation to closely related, less virulent staphylococci. They also reveal the impact that the various molecular tools that we have available (e.g., whole genome sequencing, defined transposon libraries, ect.) have had on our understanding of *S. aureus* pathogenesis. I am so excited for the future of staphylococcal research as we continue to discover the metabolic and regulatory mechanisms that allow *S. aureus* to be such a cunning pathogen.
